# Towards the year 2049: The next 25 years of occupational health and safety research

**DOI:** 10.5271/sjweh.4200

**Published:** 2024-12-01

**Authors:** Annina Ropponen, Reiner Rugulies, Alex Burdorf

**Affiliations:** 1 Finnish Institute of Occupational Health, Helsinki, Finland; 2 Division of Insurance Medicine, CNS, Karolinska Institutet, Stockholm, Sweden; 3 National Research Centre for the Working Environment, Copenhagen, Denmark.; 4 Section for Epidemiology, Department of Public Health, University of Copenhagen, Denmark; 5 Department of Public Health, Erasmus Medical Center Rotterdam, Rotterdam, The Netherlands.

**Keywords:** artificial intelligence, longitudinal, mediation, review, survey

## Abstract

**Objective:**

In this discussion paper, we close our 2024 series reflecting on the successes, failures, and promises of occupational health and safety research in celebration of the 50^th^ anniversary of the *Scandinavian Journal of Work, Environment & Health* (SJWEH). This paper aims to elaborate on the future of our research field.

Methods We conducted a narrative review of lessons learned in the series, examining insights gained and key takeaways. Additionally, we explored the current and anticipated agendas of major institutions, including the World Health Organization and the European Union, on occupational health and safety, as well as potential developments in the academic publishing industry.

**Results:**

Occupational health and safety research has significantly evolved over the last 50 years, emphasizing longitudinal study designs, enriching observational data with registry-based information, and expanding the scope of hazardous determinants impacting workers' health. Novel statistical approaches have further enabled researchers to address complex associations, such as mediation effects, and to strengthen causal inference in observational studies. At the same time, the publishing business is changing rapidly, with artificial intelligence poised to reshape both research practices and the landscape of academic publishing.

**Conclusion:**

In the changing landscape of research and academic publishing, our goal is for SJWEH to continue to be a leading source of high-quality research dedicated to protecting and improving workers’ health. We are curious and excited to see where all these current and anticipated changes will lead in the years to come.

With this article, we close out our series of discussion papers reflecting on the successes, failures, and promises of occupational health and safety research in celebration of the 50-year anniversary of the *Scandinavian Journal of Work, Environment & Health* (SJWEH). In reflecting on the future of our research field, it would have been obvious to choose a title that is looking ahead to the next 50 years. However, we reduced this expected period by half, as we wanted to have at least a theoretical chance to be still around for the next anniversary celebration.

So, how will occupational health and safety research look in 2049 when we celebrate the 75^th^ anniversary of the Journal? The honest answer is, of course, that we do not know. The *bon mot* that “prediction is very difficult, especially if it’s about the future” (allegedly coined by the Danish physicist Niels Bohr, although the German comedian Karl Valentin, the US-American writer Mark Twain, the British politician Winston Churchill, and others have also been in the conversation) is only too true. Just think about how the world looked 25 years ago. In 1999, the fall of the Soviet Union and their allies in Eastern Europe had happened less than ten years earlier, and the public health community was still intensively discussing the public health catastrophe in these countries, including the unprecedented and dramatic decline in life expectancy in Russia (1, 2). The People’s Republic of China – a country that had brought a substantial part of its population out of poverty but that was far from becoming an economic superpower and challenging the leading industrial economies in the world – was a place of cheap labor and on its way to becoming the “workshop of the world” (3). Francis Fukuyama’s book, *The End of History and the Last Man*, claimed the final and irreversible triumph of liberal Western democracies over any other social formation (4) and was still praised by many as the most accurate description of the world. Nokia was the best-selling mobile phone company in the world (5) and smartphones did not yet exist. Blockbuster was a very profitable business.

Thus, there are good reasons to take a humble approach when considering the future of occupational safety and health research. We have structured the paper into three parts:

- Lessons learned from our series on the last 50 years of occupational health and safety research;
- The current and expected agendas of major institutions, such as the World Health Organization (WHO) and the European Union (EU), on occupational health and safety; and
- The possible future of academic publishing.

## Lessons learned from our series on the past 50 years of occupational health and safety research

Among the papers published in SJWEH's 50^th^ anniversary series, some main developments of occupational exposures including asbestos, organic solvents, shift work, and psychosocial working conditions were reviewed (6–9), and some major chronic health issues, ie, musculoskeletal disorders and cancer, were debated (10, 11). These occupational health and safety topics have endured for centuries (12) and continue to be relevant today. The overview of research topics in the Journal shows a distinct shift in focus from chemical exposure to psychosocial working conditions as the most critical exposure affecting today’s working-age population (12). All these occupational exposures and health conditions are characterized by a long latency period between initial health risk warnings and reaching scientific consensus. Reaching a consensus can be complicated, with national differences and persistent challenges around uncertainty in research. This uncertainty makes it difficult to pinpoint when we have gathered sufficient knowledge to be reasonably confident (13–15).

Thus, timely and reliable knowledge is essential for implementing preventive measures; if delayed unnecessarily, it may take many years to bridge the gap between scientific consensus and the development of policies and regulations.

Good examples – such as indications on how national-level recommendations on working hours in the healthcare sector can affect shift scheduling (16, 17) – or early identification and reaction for new exposures such as carbon nanotubes (18) should be encouraged. Further, as legislation is the final step in a long and complex process, we should know more about how legislation will contribute to better occupational health and safety. As an example, asbestos and organic solvents preventive action was initially driven by researchers and social partners (6, 9). Legislation then played a crucial role in formalizing these control measures to reduce or eliminate hazardous exposures.

However, given our current understanding of psychosocial factors (7), evidence exists that national regulatory policies could promote company action plans. Consequently, these organizational-level actions could lead to preventive potential.

For decades, surveys have been the only effective way of gathering large-scale data to estimate the physical demands of work. Nowadays, various options for digital data exist, such as employer-owned register data of working hours for precise estimates of work loading due to working times (19), which can be further linked with precise data on occupational injuries (20, 21) or even with patient data to constitute further workload estimates at the ward level (22). While being a unique and new avenue for research on occupational health and work load, such vast data pools, which sometimes can even include survey data (23), have even provided possibilities to apply machine learning methods (24). In the next few years, opportunities will expand rapidly on how to use digital data for the development of advanced risk assessment tools and prognostic measures for promotive and preventive purposes in occupational health. Their use in research to provide evidence-based guidance and inform managerial decisions may also shape how existing digital data is used. Improved use of routine organizational data could reduce the need for personnel surveys or other more traditional data gathering means (23).

The assumption is that, in the long run, the same or improved information could be obtained through automatized systems from routine management in companies, occupational health services, and hospitals. However, surveys might be still needed for some specific, in-depth information such as perceived stressors and injustice at work. These kind of large, digital datasets would also be easily compatible with modern data acquisition methods, ie, real-time data from wearable technologies such as wrist- or finger-held heart rate monitors or muscle activation measurement (25, 26) devices. This ecological momentary assessment will provide researchers with much richer data that will deepen our knowledge of physical and psychosocial loading at work and lead to interesting opportunities for personalized feedback on how work influences worker’s health (27, 28).

Most workers today face a complex mixture of exposures that may affect their health. This is illustrated in Global Burden of Disease (GBD) studies, which continue to elaborate on various exposures and factors that affect disease risk such as stroke (29), while also elaborating on clusters of risks across the globe (30). Despite their evident value in gathering epidemiological evidence, we still lack sufficient knowledge about the mechanisms linking exposures or risk factors to outcomes such as health and wellbeing, as well as an understanding of potential mediating or moderating factors (31).

While meta-analyses often fail to address mechanisms, they frequently suggest the need for such studies (32). Following a recent paper on toxicology, mechanism studies would be merited even after decades of research (33). A challenge, however, is that mechanistic-oriented studies traditionally focus on single exposures, whereas the reality of current workplaces shows a myriad of types of exposure and also associated biological responses. Thus, further research should address this complexity by applying a less piecemeal focus on the association between exposure X and health outcome Y, incorporating systems thinking into the analytical approach (14, 34). This could be aligned with potential new methods that would enable the investigation of complex associations, such as neural network studies (35, 36). As our understanding of occupational health and safety continues to evolve, it calls for evaluations not only at the individual employee level but also assessments that consider structural factors at both the workplace and societal levels (37). Occupational health and safety research could be inspired by life course epidemiology (38), acknowledging that exposure operates across an individual’s life course and even across generations and that the traditional workplace exposures interact with social and physical environments in society. This requires study designs that focus on “additive effects” or an accumulation model, as well as the temporal sequence and key events in exposure–response relationships.

Thus, a final link in the chain that precedes the outcome is needed, the so-called “trigger effect” that describes a scenario where only this last link has a significant impact on the outcome. Thus, even longitudinal designs that provide a further understanding of temporal associations could be elaborated with more complex assumptions.

## Current and expected agendas of major institutions on occupational health and safety

The WHO has defined occupational health as an area of work in public health that aims to maintain and promote workers’ health and working capacity; improve working conditions and the working environment to become conducive to safety and health; develop work organization and working cultures that should reflect essential value systems adopted by the undertaking concerned, including effective managerial systems, personnel policy, principles for participation, and voluntary quality-related management practices (39). The EU has a similar definition (40). Furthermore, the science and practice of occupational health involves several disciplines, eg, occupational medicine, nursing, ergonomics, psychology, and safety research. Although occupational health has been a target of research for centuries, the transformation of the world and evolving work environment has challenged occupational health not only from a research perspective but also through legislation, interventions, and actions (40). A key example of these very rapid transformations is how climate change will affect workers’ health and wellbeing (41) in terms of rising temperatures compounding existing occupational hazards in many occupational sectors. Urbanization trends, both in relation to climate change and independently, also influence self-employment patterns (42). As a third example, digitalization has expanded the digital economy (eg, platform work) while also increasing opportunities for telework. These trends have challenged existing compensation systems for work-related musculoskeletal disorders, which could increase in the upcoming years due to the continuous transformation of working conditions (43).

To summarize this brief overview of occupational health and the changing world of work, OECD’s website of current work-life trends is worth mentioning as a valuable source of information (44).

## The future of the academic publishing

Technological advancements have changed academic publishing profoundly and will continue to do so in the future. A major change at the beginning of the 21^st^ century was the shift from paper to electronic journals. In the old days, authors sent heavy envelopes with several copies of the manuscripts by postal mail to the editors, who then forwarded the copies to the reviewers, who then returned the copies with their remarks to the editors. At the end of this process, a printed journal was published, which was sent by postal mail to libraries and other subscribers. Articles of interest were then photocopied and stored in physical folders.

Many researchers' offices are still filled with folders containing countless papers, often organized with a specific filing system to ensure each document can be easily located. But an increasing number of offices, particularly those of younger researchers, today have no trace of papers, with everything stored electronically and read on computer screens, tablets, or mobile phones.

That research journals are today published either as hybrid products (both paper and electronic versions) or purely electronically has deeply changed the publishing business. With printing costs no longer being an issue, the number of journals has exploded. *The Lancet*, for example, is no longer just one of the oldest medical journals, it has evolved into a publishing group with over 20 specialty journals. We have also seen a rise in predatory journals, money-making machines that use either no or pseudo peer-review and will publish anything as long as publication fees are paid (45). Today, researchers no longer need to worry that their papers might not be published. Anything can and will be published somewhere, as we are reminded daily by the countless emails from strange journals that ask us to submit our papers and guarantee quick publication. The result is an unprecedented level of “research waste”. It is not without irony that Douglas Altman’s famous philippic against research waste, entitled *The Scandal of Poor Medical Research* and published 30 years ago, appears from today’s perspective as a message from the good old times (46).

There is, however, always another side of the coin. The widespread emergence of electronic journals and the ability of authors to share their research publicly by uploading manuscripts to the internet, through preprint repositories or on personal websites, is placing significant pressure on traditional journals—from large publishing houses to smaller independent journals like SJWEH. This is not necessarily a bad thing. There is pressure to be better than the competition in meeting authors' needs, conduct faster peer-reviews and, perhaps most importantly, promote the movement towards open science, ensuring research can be read without barriers (47). At SJWEH, we decided to make the journal gold open access in January 2021, publishing all papers under the liberal Creative Commons CC-BY 4.0 license, which allows for the sharing and adaption of material if proper credit is given to the source (48). This decision was motivated by our commitment to the open science movement that prohibits keeping research findings behind paywalls (44). We are aware that this decision has also meant a shift in publishing costs from subscribers (usually libraries and research institutions) to the authors of the articles. Consequently, some authors, who do not have funding to pay the article processing charge (APC), will not publish in our journal and this we regret. To accommodate the needs of authors from low and middle-income countries, we wave the APC for papers with a first author from a low and lower-middle income country, and we give an 80% discount on the APC for papers with a first author from an upper-middle income country.

Calls have also been made for more radical changes in the publication business, including replacing academic journals with “a decentralized, resilient, evolvable network that is interconnected by open standards and open-source norms under the governance of the scholarly community” (49). While these calls and their main motivation – the dissatisfaction with a publication business that is dominated by a few corporate publishers – is not new, technological advancements might make alternative models today more easy to imagine than they probably were ten years ago. One can be certain though that corporate publishers will not leave the field without a fight. As a small, non-corporate, not-for-profit research journal, we can only watch, with interest, from the sidelines as this fight plays out.

When discussing technological advancements, one must speak, of course, about the role of artificial intelligence (AI) in research and publishing. During the last 12 months, many journals have updated their author instructions on the use of AI, eg, how to address the use of AI in the paper and whether ChatGPT and colleagues should be acknowledged as co-authors. These updates are made with the understanding that AI will continue to evolve rapidly, reaching levels beyond what we can currently envision. One scenario could be that researchers upload raw data to AI, the AI then analyzes the data and writes the paper, sends the paper to the journal, where it is reviewed by AI editors and reviewers who then send the paper back to the AI author, who revises the paper and send it back to the AI editors who then eventually publish the paper. This whole process of analyzing data and writing, submitting, reviewing, revising, re-reviewing, and finally publishing the paper might only take a few nanoseconds, when everything is done by AI, compared to the many months it takes today when done by humans. While this might sound a bit farfetched, the discussion about such a future publication process is already underway. In a recent paper in Nature Human Behaviour entitled *The Future of Academic Publishing*, one of the authors, Patrick Mineault, described this AI-driven research as “cheaper, faster and more interesting” and that “our ability to produce science might accelerate faster than our ability to review it and act upon it” (50). However, according to the article, this would not be a major problem, as research would no longer be read primarily by humans but by AI. The authors further elaborate: “If scientific research is mostly read by machines, the question arises of whether it is relevant to package it into a single coherent narrative that is adapted to the limitations of human cognition. This seems like a lot of busywork for scientists. We could unbundle scientific research from the constraints of journal formatting, as suggested by Neuromatch Open Publishing. In this view, research will be a living compendium of code, datasets, graphs, and narrative content — remixable and always up to date.” In this scenario of the future, AI would not only replace authors, editors, and reviewers but also the readers. This an interesting and indeed radical thought that can be considered either utopian or dystopian, depending on both one’s conviction and affection for what Mienault disdainfully labels “busywork for scientists”.

## Concluding remarks

In occupational health and safety research, we are well-equipped to respond to the need for multidisciplinary research with new methods that can address the complexity of workplace exposure. It is to be expected that causal inference considerations in observational studies will become more and more important within the complexity of multi-layered risk factors at work, acknowledging the intersectionality of work with the social and physical context of society. Decades of advancements in data collection methods, study design improvements, and the application of state-of-the-art statistical techniques have greatly benefited the occupational health community. However, the landscape of research and practice is now changing rapidly.

In this rapidly changing world, our constant goal is for SJWEH to remain a scientific journal committed to publishing high-quality research that protects and improves workers' health. We are curious and excited to see where all these current and anticipated changes in research, practice, and academic publishing will lead in the years to come.
